# The importance of event-based surveillance for preparedness and response in future respiratory pandemics

**DOI:** 10.7189/jogh.11.03098

**Published:** 2021-08-21

**Authors:** Saverio Bellizzi, Luca Cegolon, Luciano Bubbico, Salvatore Ferlito, Gabriele Farina, Giuseppe Pichierri

**Affiliations:** 1 Medical Epidemiologist, Independent Consultant, Geneva, Switzerland; 2 Local Health Unit N.2 “Marca Trevigiana”, Public Health Department, Treviso, Italy; 3 Department of Sensorineural Disabilities, INAPP/Italian Institute of Social Medicine, Rome, Italy; 4 Department of Surgical Medical Sciences and Advanced Technologies, School of Medicine, University of Catania, Catania, Italy; 5 University of Sassari, Sassari, Italy; 6 Microbiology Department, Kingston Hospital NHS Foundation Trust, London, UK

MERS-CoV was first recognized in 2012 as the cause of severe lower respiratory tract infection in humans and by the end of April 2021, a total of 2574 laboratory-confirmed cases and 886 associated deaths (case-fatality ratio 34.4%) were reported globally [1]. On the other hand, the current COVID-19 pandemic has already caused almost 170 million cases and 3.5 million deaths worldwide [2].

Though several low- and middle-income countries have a robust case-based national integrated disease surveillance system, the threat of emerging respiratory pathogens illustrates a need for early detection of unusual health events, or event-based surveillance, in the country. Specifically, in the Middle East, the threat of Middle East Respiratory Syndrome Coronavirus (MERS-CoV) prompted several Ministries of Health over the last decade to implement a country-wide event-based surveillance, with a focus on unusual respiratory events [3].

*Event-based surveillance (EBS)* is the organized collection, monitoring, assessment, and interpretation of mainly unstructured, ad hoc information regarding health events or risks, which may represent an acute risk to human health. EBS systems rely on the immediate reporting of signals of potential events. EBS systems are designed to detect unusual and new events that are not specifically captured in traditional case-oriented indicator-based surveillance systems [4].

Specifically, signals are data and/or information on events considered to represent a potential acute risk to human health, such as an outbreak of communicable diseases. Signals may consist of reports of unusual cases or deaths (individual or aggregated), an unusual pattern of disease, a sudden increase of disease, a change in the clinical presentation of a disease, etc.

Unexplained severe acute respiratory illness that occurs in a health care worker who takes care of patients with respiratory illness, severe acute respiratory illness in humans that is associated with illness in animals, and changes in expected patterns of acute respiratory illness, including increase in apparent mortality or unexplained deaths, are just few examples of unusual respiratory signals that can be required for immediate notification.

Building on the experience from SARS in 2003, MERS-CoV in 2014 and COVID-19 (third coronavirus outbreak emergence that has occurred over the past two decades), a novel respiratory virus could be detected through a comprehensive EBS using scenarios such as: three individuals in the same area who are severely ill, with two of them rushed to a hospital with severe respiratory illness, or the animal health sector sharing reports of sudden sickness and deaths of poultry in a community and in an animal market, or the national public health laboratory detecting an unsubtypeable influenza virus from hospitalised patients [5]. Additional useful elements can be provided by media reporting of increasing public anxiety over a number of hospitalised cases from one municipality or a school reporting several children absent with severe respiratory illness [5].

**Figure:**
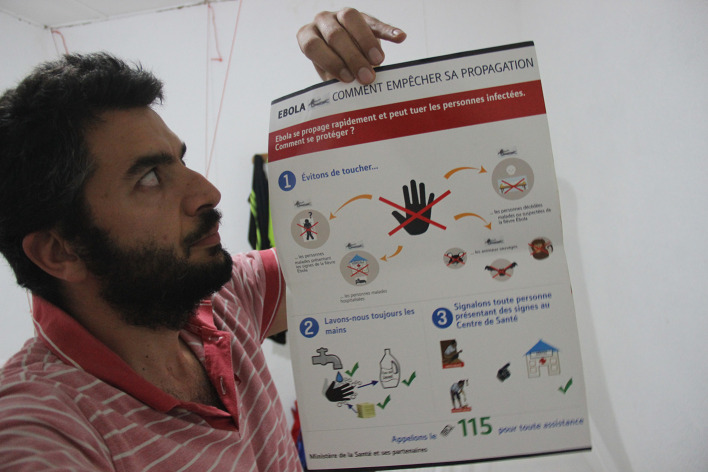
Photo: Surveillance and risk communication and community engagement during the Ebola outbreak in 2014 (from Saverio Bellizzi’s own collection, used with permission).

Not all signals are indications of outbreaks, but each signal must be carefully and systematically verified. When a signal is verified, it becomes an “event”. Events should trigger or prompt some level of response, including further inquiry, investigation, and containment. Therefore, the early detection of events through EBS is intended to initiate early action through appropriate response mechanisms, and ultimately mitigate public health consequences, and prevent outbreaks.

The reporting of unusual respiratory signals enables early detection of unusual respiratory pathogens/diseases prioritized by different Ministries of Health, which might include avian influenza, MERS-CoV, and other emerging respiratory pathogens. In order to ensure efficiency and effectiveness, signals are required to be notified immediately, or within 24 hours, upon recognition by clinicians, nurses, and focal points in all hospitals.

Response to respiratory event is a continuous cycle that should include all the different levels of the health system structure in a country: as indicated by Balajee et al., as an event evolves and information becomes available, evaluation and risk assessment processes may need to be repeated to provide a longitudinal understanding of the development and trajectory of the event [5]. In several occasions it is fundamentally important the constant collaboration with other public sectors, like the Ministry of Agriculture and Ministry of Environment, and other significant stakeholders. By actively reducing the impact of an unusual respiratory event, and preventing its spread, a rapid response will effectively and efficiently control and mitigate unusual respiratory events.

The success of EBS implementation is contingent on the early detection and reporting of signals and events through a country’s surveillance and reporting structure. Aspects like timeliness, flow of information and reporting structure should align with national surveillance. Useful framework for low- and middle-income countries (LMICs) are available, such as the one proposed by the Africa Centres for Disease Control and Prevention (CDC) which clarifies the role of local level authorities in triaging and verifying signals, and reporting events up to the intermediate-level [6]. On the other hand, all events received at the intermediate level require an assessment of risk in consultation with higher administrative levels. Intermediate-level authorities should then provide feedback about events and signals to reporters at the local level; feedback on reported signals should be given to smaller health facilities and stakeholders at the community-level by local-level authorities [6]. In light of likely future respiratory outbreaks, immediate investment on enhanced surveillance must be among the priority items for advanced preparedness and readiness at country and sub-country levels. Rapid risk management of public health events can reduce or prevent disease in affected populations, as well as associated negative social and economic consequences.
